# Health Risk Assessment Related to Waterborne Pathogens from the River to the Tap

**DOI:** 10.3390/ijerph120302967

**Published:** 2015-03-10

**Authors:** Pauline Jacob, Annabelle Henry, Gaëlle Meheut, Nadine Charni-Ben-Tabassi, Valérie Ingrand, Karim Helmi

**Affiliations:** Veolia Recherche et Innovation, Immeuble le Dufy, Centre de recherche de Saint Maurice, 1 place de Turenne, 94417 St Maurice Cedex, France; E-Mails: annabelle.henry@veolia.com (A.H.); gaelle.meheut@veolia.com (G.M.); nadine.charni-ben-tabassi@veolia.com (N.C.-B.-T.); valerie.ingrand@veolia.com (V.I.); karim.helmi@veolia.com (K.H.)

**Keywords:** waterborne pathogens, health risk assessment, water, fecal pollution indicator, land use

## Abstract

A two-year monitoring program of *Cryptosporidium parvum* oocysts, *Giardia duodenalis* cysts, *Escherichia coli*, *Clostridium perfringens* spores and adenovirus was conducted in three large rivers in France used for recreational activities and as a resource for drinking water production. Fifty-liter river water and one thousand-liter tap water samples were concentrated using hollow-fiber ultrafiltration and analyzed by molecular biology or laser-scanning cytometry. In order to evaluate watershed land use influence on microorganism concentration changes, occurrence and seasonality of microorganisms were studied. The highest concentrations of protozoan parasites and *C. perfringens* were found for one of the three sites, showing a high proportion of agricultural territories, forests and semi-natural environments, which may be partly attributable to soil leaching due to rainfall events. On the contrary, the highest concentrations of adenoviruses were found at the two other sites, probably due to strong urban activities. Health risk assessment was evaluated for each waterborne pathogen regarding exposure during recreational activities (for a single or five bathing events during the summer). The calculated risk was lower than 0.5% for parasites and varied from 1% to 42% for adenovirus. A theoretical assessment of microorganism removal during the drinking water treatment process was also performed, and it showed that an absence of microorganisms could be expected in finished drinking water. This hypothesis was confirmed since all tested tap water samples were negative for each studied microorganism, resulting in a risk for drinking water consumption lower than 0.01% for parasites and lower than 0.5% for adenovirus.

## 1. Introduction

River water is widely used as a resource for drinking water production and for recreational activities. The number of bathing sites and sport practices in aquatic environment is increasing and as a result the management of sanitary risks requires particular attention. Indeed, river water is usually contaminated by bacteria (e.g., *E. coli*, *C. perfringens*), viruses (e.g., adenovirus) and pathogenic protozoa (e.g., *G. duodenalis*, *C. parvum*) that can pose significant health problems.

*G. duodenalis* and *C. parvum* are intestinal protozoan parasites that can infect humans and other mammalian species [1,2]. Ingestion of *G. duodenalis* can lead to severe, chronic or asymptomatic infections. When the infection is symptomatic, giardiasis causes diarrhoea, nausea and anaemia [1]. *C. parvum* is responsible of cryptosporidiosis, causing symptoms ranging from benign to severe diarrhoea with several possible complications. Numerous pathways of both parasites’ transmission exist, including animal-to-human, person-to-person and waterborne contamination [3,4]. Human contamination may occur because *G. duodenalis* and *C. parvum* are able to survive in the environment for long periods of time [5,6], with usually a higher persistence for *C. parvum* oocysts in comparison with *G. duodenalis* cysts [7,8].

Fifty-four human adenovirus serotypes have been identified, which can cause infantile gastroenteritis and symptomatic infections in numerous organs, including respiratory organs, eyes, the gastrointestinal tract, central nervous system, and sexual organs [9,10]. Human adenovirus types 40 and 41 are often related with infant gastroenteritis, and type 4 is mainly linked to respiratory diseases [11,12]. Adenovirus transmission occurs by both a fecal-oral route and inhalation of aerosols [13]. They are persistent in feces, urine and respiratory secretions of contaminated persons [9]. Humans are not their only host, as animal-specific adenoviruses infect a wide range of animals [14]. Adenoviruses were included in the “Candidate Contaminant List” (http://water.epa.gov/scitech/drinkingwater/dws/ccl/ccl3.cfm) as part of the Safe Drinking Water Act by the U.S. Environmental Protection Agency (USEPA), in 1998 [15]. They were included in this list in particular for their UV light resistance, and because their survival characteristics during water treatment are not yet fully understood, as well as their public health implications.

There is no standard for acceptable adenovirus levels in surface water, but some studies showed that their presence could be indicative of fecal pollution, according to relationships between fecal indicator organisms and adenovirus concentrations [16], although contradictory data exist [17]. The Virobathe project (http://www.virobathe.org/) showed that this virus is a relevant candidate indicator organism. Adenoviruses seem to be particularly efficient at distinguishing human and non-human fecal pollution in microbial source tracking applications [18,19]. It was shown that adenovirus may be discharged into the environment with sewage effluent [19], and outbreaks associated with recreational waters have been linked with their presence in polluted waters [16].

*E. coli* is found in high concentration in all mammalian feces. It has been chosen as a water safety biological indicator and as such it was included in drinking water regulations (Council of the European Union, Council Directive 98/83/EC on the Quality of Water Intended for Human Consumption). *E. coli* can survive in drinking water for several weeks, depending on environmental conditions [20]. *E. coli* is also used as a fecal pollution indicator for recreational bathing waters in Europe (Directive 2006/7/EC of the European Parliament and the Council Concerning the Management of Bathing Water Quality and Repealing Directive 76/160/EEC).

Concerning humans, *C. perfringens* causes food poisoning with the most often observed symptoms being diarrhoea, necrosed enteritis with hemorrhagic diarrhea and necrosis of the intestinal wall. *C. perfringens* is a very ubiquitous telluric bacteria which is widely spread in environment (soil, sediments, sewage, manure, corpses, vegetable surfaces…) [21]. Regarding animals, it causes numerous severe diseases, in particular enteritis, enterotoxemia and dysentery. *C. perfringens* spores’ long life is the major impediment to their use as a fecal contamination indicator [20]. However, to date, standards for acceptable levels of *C. perfringens* in surface water have not yet been established, although it is included as a facultative indicator in the European Directive 98/83/EC (Annex I, part C) because it is thought to be a fecal pollution indicator.

In this context, the aim of our study was to investigate microorganism occurrences in three river waters utilized both for recreational activities and as resources for three drinking water treatment plants, confronting pathogens and fecal indicator concentration levels with land use data, through a two-year monitoring campaign. Tap water samples, served from a drinking water treatment plant fed by one of the three resources, were also analyzed.

Health risks related to *C. parvum*, *G. duodenalis* and adenovirus by accidental ingestion of river water during recreational activities, were assessed through exponential dose-response models. The three drinking water treatment plants must produce and distribute every day a water quality in compliance with microbiological standards. Efficiency of drinking water treatment process to remove microorganisms was theoretically estimated, focusing on the drinking water treatment plant which produced the analyzed tap water. This estimation took into account measured concentrations at the corresponding river water resource and log-removal levels related to each microorganism. Finally, health risk was assessed for drinking water consumption, on the basis of the results obtained on the tap water samples.

## 2. Experimental Section

### 2.1. Sampling Design and Simultaneous Concentration of Parasites, Bacteria and Viruses

The sampling campaign was performed over two years (2012 and 2013) at sites identified as A, B, C (three French large rivers) and D (tap water served by the drinking water treatment plant fed by source C). The drinking water (collected directly at the tap at site D) had travelled approximately 10-km between the drinking water treatment plant and the tap, through assorted pipes composed of cast iron, steel and cement. River grab water samples were collected according to the season at the plant inlet. Carboys were then sent by shuttle the same day and processed for filtration within 24 h.

Fifty liters of river water, or one thousand liters of drinking water (dechlorinated online using Akdolit^®^ Hydrochlorex granules), were filtered through a disposable polysulfone hollow-fiber ultrafilter module (HF80S, Fresenius Medical Care, Fresnes, France). Water was pushed through the hollow fibers using a peristaltic pump, with a flow rate of 3 L per minute. The filter presents an exclusion size of 25–30 nm allowing the simultaneous collection of different microorganism groups (parasites, bacteria, viruses…) from large volumes of water within a short period of time (30 min for river water and about 6 h for drinking water). The filter cartridge was capped at the opposite end, forcing water through the pores of the fibers in a dead-end filtration path, without recirculation of water. Water and particles smaller than the pore size were discarded while microorganisms were retained in the fibers.

After filtration, the microorganisms trapped within the fiber cores were recovered by backflushing in the reverse direction (from the outside to the inside of the fibers) with one liter of eluting solution composed of phosphate buffered saline 0.01M (P3813, Sigma Aldrich France, Lyon, France), 0.5% Tween 80 (P4675, Sigma Aldrich France), 0.01% Sodium hexametaphosphate (305553, Sigma Aldrich France) and 0.1% antifoam B (A5757, Sigma Aldrich France) pushed by the peristaltic pump and the concentrate was collected in a glass beaker. The concentrate was then preserved during 48 h maximum, at 4 °C, before analysis. In river water, recovery rates for the concentration method were greater than 100% for viruses, 60% for *E. coli*, 55% for *C. perfringens* and 89% for *C. parvum*; in drinking water, yields were 64%, 79%, 84% and 96%, respectively.

### 2.2. Escherichia Coli Detection

Samples collected during the two-year campaign were analyzed for *E. coli* by quantitative-Polymerase Chain Reaction (q-PCR) detection, using 150 mL of the concentrate volume, representing 7.5 L of the initial river water sample or 150 L of the initial drinking water sample. The filtration of the concentrate was fractionated over three membranes (Pall Life Sciences GN-6 Metricel Grid 47 mm, 0.45 µm) to avoid clogging. Membranes were then collected in 9 mL of Nuclisens lysis buffer (280134, Biomérieux, Craponne, France), for DNA extraction using a Nuclisens Minimag work station. Membranes were mixed during two minutes and conserved at −80 °C until extraction. Samples were then processed using the Nuclisens Magnetic Extraction Reagents kit (200293, Biomérieux), according to the manufacturer’s instructions.

Detection of *E. coli* was based on the *sfmD* gene, encoding a putative outer membrane export usher protein [22], using the pair of oligonucleotide primers *SfmD forward* 5' ACT GGA ATA CTT CGG ATT CAG ATA C 3' and *SfmD reverse* 5' ATC CCT ACA GAT TCA TTC CAC GAA A 3' and the probe *SfmD probe* 5'-6-FAM-CAG CAG CTG GGT TGG CAT CAG TTA TTC G-TAMRA-3'. For the q-PCR step, 5 µL of extracted DNA were added to 20 µL of Absolute Blue Master (Thermo Scientific, Illkirch, France) containing 2× reaction buffer, 500 nM of each primer and 200 nM of probe. Diethylpyrocarbonate (DEPC) water (AM9906, Life Technologies, Saint Aubin, France) was used as negative control. The PCR assay was performed with a Light Cycler 480 (Roche Diagnostics, Meylan, France). The activity of the DNA polymerase was released by heating at 95 °C for 15 min. Samples were then submitted to 45 cycles (15 s at 95 °C and 45 s at 62 °C). Real time fluorescence was measured at the end of the extension step, every cycle and analyzed by the Light Cycler 480 software. Each extract was analyzed undiluted, log diluted and twice log-diluted (5 µL in 45 µL of DEPC water), in triplicates. Determined concentrations were expressed in genomic units (GU) per filtered volume. The detection limit (LOD) of the PCR was 10 GU per reaction.

### 2.3. Clostridium Perfringens Spore Detection

Samples collected during the two-year campaign were analyzed using 150 mL of the concentrate volume, corresponding to 7.5 L of the initial river water sample or 150 L of the initial drinking water sample. The concentrate volume was heated at 75 °C during 18 min (vegetative form destruction and spore germination) and then filtered on three membranes (Pall Life Sciences GN-6 Metricel Grid 47 mm, 0.45 µm). Membranes were then collected in 9 mL of Nuclisens lysis buffer, for DNA extraction using Nuclisens Minimag work station (Biomérieux), with the same procedure as for *E. coli*.

Detection of *C. perfringens* was based on the alpha toxin gene [23], using the pair of oligonucleotide primers *CPA TQ1* 5' CTA GAT ATG AAT GGC AAA GAG GAA ACT A 3' and *CPA TQ2* 5' TTA GCA GGA TGA TAT GGA GTA TCT ATA TCT C 3' and the *CPA TQP probe* 5'-FAM-AAA CAA GCT ACA TTC TAT CTT GGA GAG GCT ATG CAC TAT T-TAMRA-3'. For the q-PCR step, 5 µL of extracted DNA were added to 45 µL of Probes Master Mix Roche (Roche Diagnostics) containing 2× reaction buffer, 300 nM of each primer and 100 nM of probe. DEPC water was used as negative control. The PCR assay was performed with a Light Cycler 480. The activity of the DNA polymerase was released by heating at 95 °C for 10 min. Samples were then submitted to 45 cycles (15 s at 95 °C and 1 min at 60 °C). Real time fluorescence was measured at the end of the extension step, every cycle and analyzed by the Light Cycler 480 software. Each extract was analyzed undiluted and log-diluted (5 µL in 45 µL of DEPC water), in triplicates. Determined concentrations were expressed in GU per filtered volume. The LOD of the PCR was 2 GU per reaction.

### 2.4. Human Adenovirus Detection

Samples were analyzed using 150 mL of the concentrate volume, corresponding to 7.5 L of the initial river water sample or 150 L of the initial drinking water sample. The concentrate volume was filtered in three times on two membranes stacked, one for the sample prefiltration and the second for the filtration (Glass Fiber Prefilter, AP2004700 reference, Millipore and nylon membrane, NM 04701045SP reference, CUNO 3M). Both membranes were then collected in 15 mL of Nuclisens Lysis Buffer (Biomérieux), for DNA extraction using Nuclisens Minimag work station (Biomérieux), with the same procedure as for *E. coli*.

Detection of all species of human adenoviruses was based on the Hexon gene, using the pair of oligonucleotide primers *AD forward* 5'-CWT ACA TGC ACA TCK CSG G-3' and *AD reverse* 5'-CRC GGG CRA AYT GCA CCA G-3' and the *ADP probe* 5'-FAM-CCG GGC TCA GGT ACT CCG AGG CGT CC-TAMRA-3'. For the q-PCR step, 5 µL of extracted DNA were added to 20 µL of Probes Master Mix Roche (Roche Diagnostics) containing 2X reaction buffer, 900 nM of each primer, 200 nM of probe and 0.025 µg/µL of T4 Gene 32 Protein (10972983001, Roche Diagnostics). DEPC water was used as negative control. The PCR assay was performed with a Light Cycler 480. The activity of the DNA polymerase was released by heating at 95 °C for 5 min. Samples were then submitted to 45 cycles (15 s at 95 °C and 1 min at 60 °C). Real time fluorescence was measured at the end of the extension step, every cycle and analyzed by the Light Cycler 480 software. Each extract was analyzed undiluted and log-diluted (5 µL in 45 µL of DEPC water), in triplicates. Determined concentrations were expressed in GU per filtered volume. The LOD of the PCR was 0.1 GU per reaction.

### 2.5. Cryptosporidium Parvum Oocyst and Giardia Duodenalis Cyst Detection

Two hundred milliliters of concentrate, corresponding to 10 L of the initial river water sample or 200 L of the initial drinking water sample, were centrifugated at 1250 g for 30 min at 4 °C. The supernatant from each tube was then aspirated and discarded. The pellet was resuspended into 10 mL of Phosphate Buffered Saline 0.01 M (P3813, Sigma Aldrich France) and transferred in a Leighton tube, based on the standard method NF T 90-455-July 2001. Anti-*G. duodenalis* and anti-*C. parvum* beads from Immuno-Magnetic Separation (IMS) kit (Dynabeads *Cryptosporidium Giardia* Combo kit, Invitrogen Dynal AS, Oslo, Norway) were added according to the manufacturer’s instructions. For analysis, the entire eluate (100 µL) resulting of the IMS was transferred to adapted well slides and dried at 36 °C in a dry off oven. The parasites were then fixed by addition of 50 µL of methanol.

The slides were then stained with the *Cryptosporidium Giardia* detection kit on slide (Chemunex, Ivry sur Seine, France) following the manufacturer’s instructions. Quantification of the parasites was carried out by a screening of the slides with a solid-phase cytometer device (ChemScan RDI, AES-Chemunex, France), which consists in a laser-scanning unit (488-nm argon laser) able to scan a 25-mm diameter membrane filter within 3 min. Determined concentrations were expressed in (oo)cysts per volume. The LOD for this method was 1 (oo)cyst per analysed volume.

### 2.6. Physicochemical Analysis

River water temperatures were measured at the three sites A, B and C and data regarding flow rate and rainfall were collected. For this study, cumulative rainfall data were taken into account during three days prior the sampling day, in order to be more representative regarding the distance and the flow rate.

### 2.7. Health Risk Assessment

For swimmers, the risk assessment was based on the probability of microorganisms to generate illness for two groups of swimmers: adults with primary contact and children with primary contact. This methodology consists in three main points : the potential for presence of pathogens, the potential for health effects and the risk characterization [24]. The risk of infection related to *C. parvum*, *G. duodenalis* and adenovirus for one-time swimming was estimated using a previously described dose-response model [25,26]. This exponential model Equation (1) allow to relate the probability of becoming infected (*P_inf_*) to the mean dose (number of microoorganisms inhaled or ingested) (N):

(1) $$P_{inf}=1-e^{-rN}$$

Protozoa and viruses tend to follow this model, for which r is a constant describing the probability of infection by the microorganism, inhaled or ingested (*r* = 0.0042, 0.0199 and 0.4172 for *C. parvum*, *G. duodenalis* and type 4 adenovirus, respectively) [9,25,26,27,28]. In this model, microorganisms follow a Poisson distribution (random distribution within the water volume). The risk of infection was also calculated considering five bathing episodes, following this second model Equation (2) (qmrawiki.msu.edu):

(2) $$P_{inf}=1-(1-\;\left(1-e^{-rN}\right)){\;}^{5}$$

The first model Equation (1) was also used to evaluate the risk associated with drinking water consumption, based on the results obtained at site D, on tap water samples. For the calculation, an average consumption of 1 L of tap water per day was taken into account.

The risk calculation was performed on non-bacterial pathogenic microorganisms which are more persistent in the environment and resistant to treatments. In this study *E. coli* and *C. perfringens* were only considered as indicators.

## 3. Results

A total of 32 river water samples (eight per site) and eight tap water samples were collected during this two-year study. Fifty-liter river water and one thousand-liter tap water samples were concentrated using hollow-fiber ultrafiltration and were analyzed for the presence of parasites (*C. parvum* and *G. duodenalis*), indicator bacteria (*E. coli*, *C. perfringens*) and adenovirus (Table 1).

**Table 1:** Microorganism concentrations for river (50 L filtered) and drinking water samples (1000 L filtered).

Sampling Site		*C. Parvum* (n/Volume)	*G. Duodenalis* (n/Volume)	*E. Coli* (GU/Volume)	*C. Perfringens* (GU/Volume)	Adenovirus (GU/Volume)
A	Average year	12	36	1.5 × 10^4^	1.1 × 10^3^	3.8 × 10^2^
	Min year	<1	<1	<1.1 × 10^3^	1.0 × 10^2^	<7 × 10^0^
	Max year	40	95	7.1 × 10^4^	5.4 × 10^3^	1.5 × 10^3^
	Max summer	15	20	8.4 × 10^3^	2.7 × 10^2^	6.8 × 10^1^
B	Average year	3	43	1.6 × 10^4^	4.7 × 10^2^	1.3 × 10^3^
	Min year	<1	5	2.0 × 10^2^	<2.5 × 10^2^	<7 × 10^0^
	Max year	10	140	4.0 × 10^4^	2.0 × 10^3^	5.7 × 10^3^
	Max summer	5	30	8.2 × 10^3^	7.7 × 10^1^	9.0 × 10^1^
C **	Average year	1	10	8.8 × 10^3^	4.0 × 10^2^	8.8 × 10^2^
	Min year	<1	<1	<1.1 × 10^3^	<2.5 × 10^2^	<7 × 10^0^
	Max year	5	30	1.8 × 10^4^	1.7 × 10^3^	4.2 × 10^3^
	Max summer	0	20	8.5 × 10^3^	7.4 × 10^1^	3.5 × 10^2^
D *		<1	<1	<823	<1.6 × 10^2^	<1.1 × 10^1^

Notes: * For one of the eight drinking water samples a volume of 426 L was filtered; ** C used as resource for tap water D production; The overall detection limit took into account the recovery rate of the concentration method and the LOD of the detection method.

Results of the campaign showed that 10 (42%) and 21 (88%) river water samples out of 24 were positive for *C. parvum* and *G. duodenalis*, respectively. Site A was slightly more contaminated by *C. parvum* and *G. duodenalis* as five water samples out of eight were positive for both parasites, with concentrations reaching 40 and 95 (oo)cysts 50 L^−1^, respectively (Table 1). By comparison, at sites B and C, three and two samples out of eight were positive for both parasites, respectively. For *E. coli*, similar concentrations were observed at the three sites A, B and C varying in average from 8.8 × 10^3^ to 1.6 × 10^4^ GU *E. coli* 50 L^−1^ (Table 1).

Site A appears as the most contaminated by *C. perfringens*, since all eight samples were positive, with an average concentration of 1.1 × 10^3^ GU *C. perfringens* 50 L^−1^. In addition, levels were two or three times higher at this site than those observed at sites B and C (average concentrations of 4.7 × 10^2^ and 4.0 × 10^2^ GU *C. perfringens* 50 L^−1^, respectively, Table 1).

Sites B and C were most contaminated with adenovirus (seven positive samples out of eight) in comparison with site A (five positive samples out of eight). The average concentrations varied from 3.8 × 10^2^ to 1.3 × 10^3^ GU adenovirus 50 L^−1^ (sites A and B respectively). All tap water samples from site D were negative for each tested microorganism.

### 3.1. Land Use of the Watersheds

Table 2 summarizes the land use distribution found for the river sampling sites.

**Table 2:** Land use distribution for sites A, B and C.

Site Area	Artificial Territories	Agricultural Territories	Forests and Semi-Natural Environments	Wet Zones
A	26%	55%	19%	1%
B	93%	4%	3%	1%
C	83%	7%	9%	1%

Site A presents a high proportion of natural environments (agricultural territories, forest and semi-natural environments including shrubby and/or herbaceous vegetation and open spaces, with low or without vegetation), at a cumulative level about 74%. On the contrary, at sites B and C, the land use is mainly urban, with high proportions of artificial territories (urban, industrial, commercial zones and transport network, wastewater treatment plants, discharge and construction sites, green artificial spaces), reaching 93% and 83%, respectively.

### 3.2. Risk Assessment for Swimmers during Recreational Activities

The risk of infection related to *C. parvum*, *G. duodenalis* and adenovirus during recreational activities near the three river water sampling points was assessed (Table 3). It is important to note that the calculation was based on the maximum concentrations found in summer, when the health risk associated with the ingestion of contaminated surface water is the most probable, due to bathing activities. According to our results, the highest protozoan concentrations observed in summer at site A were 15 and 20 (oo)cysts 50 L^−1^ of *C. parvum* and *G. duodenalis*, respectively. During the same period, concentrations at site B reached five and 30 (oo)cysts 50 L^−1^ of *C. parvum* and *G. duodenalis* respectively, and at site C, 0 and 20 (oo)cysts 50 L^−1^ of *C. parvum* and *G. duodenalis*, respectively. The highest adenovirus concentrations observed in summer at site A, B and C were respectively 6.8 × 10^1^, 9.0 × 10^1^ and 3.5 × 10^2^ GU adenovirus 50 L^−1^ (Table 1).

**Table 3:** Calculation of the probability of becoming infected (*P_inf_*) during summer for three waterborne pathogens detected in river waters.

Microorganism	Swimmers	*Pinf* (%)			Equation Model
		Site A	Site B	Site C	
*C. parvum*	Adult	0.002	0.001	<0.001	(1)
		0.010	0.003	<0.001	(2)
	Child	0.005	0.002	<0.001	(1)
		0.023	0.008	<0.002	(2)
*G. duodenalis*	Adult	0.013	0.019	0.013	(1)
		0.064	0.096	0.064	(2)
	Child	0.029	0.044	0.029	(1)
		0.147	0.221	0.147	(2)
Adenovirus	Adult	0.904	1.194	4.616	(1)
		4.438	5.831	21.046	(2)
	Child	2.078	2.740	10.353	(1)
		9.965	12.971	42.100	(2)

Notes: (1) For one-time bathing; (2) For five bathing episodes.

Considering an average volume of 16 mL of water ingested by an adult and 37 mL by a child [29], the maximum risk of infection for the three sites A, B and C during summer was lower than 0.05% for both parasites for one-time bathing. For five baths during summer, the maximum risk of infection increases, but remains lower than 0.5%.

For an adult and one-time bathing, regardless to the site, the maximum risk of infection for adenovirus ranged from 0.904% to 4.616% and increased from 4.438% to 21.046% for five baths. For a child, it ranged from 2.078 to 10.353% for one-time bathing and from 9.965% to 42.100% for five baths.

### 3.3. Risk Assessment for Drinking Water Consumption

The probability of becoming infected (*P_inf_*) was calculated according to the first model Equation (1), considering an ingested volume of one liter of tap water per day, and taking into account the initial volume filtered of 1000 L. Considering a volume of 1 L of tap water ingested per day, the maximum risk of infection for the tap water D was lower than 0.003%, 0.02% and 0.5% for *C. parvum*, *G. duodenalis* and adenovirus, respectively.

## 4. Discussion

### 4.1. Watershed Land Use and Seasonality

Surface waters are commonly contaminated with *C. parvum* and *G. duodenalis*, due to potential fecal contamination sources like wastewater treatment plants and agricultural activities present in the sampling area [30,31]. It has been shown that high precipitation events and colder temperatures are favorable for the transfer and survival of (oo)cysts through runoff into surface waters [5,32]. Results showed that higher concentrations of *G. duodenalis* were almost systematically observed in comparison with *C. parvum* for the three sites, which was already found for surface water in previous studies [33,34]. Regarding the seasonality of microorganisms, highest occurrences of *C. parvum* were mainly measured in autumn, winter and spring and highest occurrences of *G. duodenalis* were measured in winter and spring.

*C. perfringens* is commonly found in sewage [35,36], and does not frequently occur in ruminant wildlife, but rather is mainly associated with mixed-diet and carnivorous organisms’ excreta and human sewage [37]. In the present study, *C. perfringens* was prevalent during winter time when the water temperature is less than 10 °C, suggesting that low temperatures are favorable to *C. perfringens* persistence, as for parasites [24,38,39]. This may be explained by a higher persistence of the spores at low temperature in winter [38,39], when runoff increases and with sediment disturbance [24].

The most important concentrations of parasites and *C. perfringens* spores were found at site A. This can be explained by the land use of this area, presenting agricultural lands (55% of the surface area, with probable breeding and manure spreading activities), forests and semi-natural environments (19%), in comparison with artificial territories (26%) (Table 2). Indeed, site A is surrounded by two of the largest regional natural reserves of this geographical zone, with recreational activities like horse-riding, located upstream from the sampling point. Many precipitations were observed during the sampling period at this site (especially during spring and at the end of summer, with cumulative levels reaching 10.1, 16.0 and 15.6 mm over 3 days in April, May and September respectively), in comparison with sites B and C, resulting in leaching of soils from agricultural territories or from forests and semi-natural environments. As a result, these non-point sources (diffuse pollution sources) could be an input of parasites and *C. perfringens*. Furthermore, at site A, eight wastewater treatment plants were found, located upstream from the sampling point, within a distance of 20 km (four treatment plants with a capacity comprised between 2000 and 10,000 inhabitants, and four with a capacity between 10,000 and 50,000 inhabitants), representing a probable additional pollution source for parasites and *C. perfringens*.

*E. coli* is known to be a fecal pollution indicator in bathing water. However, concentration levels did not present significative variations between the three sites during the monitoring period, with a persistent basic threshold concentration, unlike other microorganisms for which differences were found according to the land use data. As a result, this raises the question of the reliability of this indicator to detect the presence of fecal pollution in river water.

Previous studies reported a strong association between adenoviruses and sources of human fecal pollution [40]. Contamination can be related to urban discharges contaminating river waters (from wastewater treatment plants, autonomous sewer, direct discharge, swimmers…). The highest occurrence of adenovirus was measured in spring time season. Factors controlling the occurrence, survival and distribution of enteric viruses in the environment include host excretion and water temperature [18,41,42]. Indeed, maximum host excretion occurs in summer, which coincides with recreational activities and human-water contact [18,43], but their survival and infectivity is maximal during winter when temperatures are lower [27]. These observations could explain that the maximum occurrence of adenoviruses was found in a mid-temperate season like springtime.

The highest concentrations of human adenoviruses were found at site B and C, where the urban territory proportions are the most important (93% and 83%, respectively), conversely to site A (26%), for which the human adenoviruses concentrations were the lowest. As a result, the most probable sources of human adenoviruses could be the wastewater treatment plants, upstream from the sampling point. Indeed, at site C, a large wastewater treatment plant with a capacity of 3.6 million inhabitant equivalents was found at less than 5 km upstream from the sampling point.

### 4.2. Risk Assessment for Recreational Activities

This study was undertaken to evaluate the health risk associated with waterborne pathogens *C. parvum*, *G. duodenalis* and adenovirus in river waters used for recreational activities. For instance, at site A, a recreation area is located 25 km upstream from the sampling point, in one of the largest regional natural reserves of this geographical zone. The risk for swimmers was calculated based on the concentration levels measured at the sampling point, located downstream the recreation area.

Although *G. duodenalis* and *C. parvum* were prevalent from autumn to spring when the temperatures were lower, the probability of ingestion of (oo)cysts is the most important in summer, when most of the recreational activities take place. Taking into account the concentration levels obtained during summer, the calculated risk was very low for both parasites, lower than 0.5% for all sites, regardless to the considered swimmer (adult or child) or the number of bathing episodes (one or five). It is important to note that infectivity of both parasites was not assessed for ethical reasons. Consequently, laser-scanning cytometry was selected despite a potential overestimation compared to infective (oo)cysts.

In the same way, based on the concentration levels obtained in summer, the risk associated with adenovirus is clearly much more important in comparison with parasites, especially for a child at site C (>10% for one-time bathing and >40% for five bathing episodes). However, these values should be treated with caution as there is little published data available on the “r” value determination [44], and the associated risk calculation for adenoviruses was based on a very limited number of subjects in clinical trials [45]. Indeed, the r value was determined for the type 4 adenovirus (mainly responsible of respiratory diseases) and not for all types of adenoviruses. Moreover, adenoviruses were detected using a q-PCR method, which detects at the same time infectious viral particles, damaged particles and free DNA present in the sample, leading to a possible overestimation of the risk. Indeed, previous studies demonstrated that not all ingested or inhaled microorganisms reach a target site where they can initiate a response, because they may be not viable or their activity may be impaired due to host defences [44]. However, culture methods are not effective to detect all adenovirus strains and as a result PCR was chosen. Furthermore, it is important to notice that in a context of risk assessment, an overestimation is preferable rather than an underestimation which could lead to health risks.

The highest probabilities of infection by adenoviruses were found at site C. It’s interesting to notice that a recreational area was found surrounding site C, within a distance of 8 km from the sampling point, which proposes nautical activities like kayaking. At site C it would be interesting to advise a monitoring of adenovirus, taking samples directly on the site of nautical activities, allowing a new risk assessment and to attempt to identify more precisely the adenovirus pollution sources involved. On the contrary, lowest probabilities of infection by adenoviruses were found at site A, showing a recreational area with bathing activities within a distance of 25 km from the sampling point. Fortunately, site A was the less contaminated and adenoviruses were prevalent in spring, outside of the periods of intensive bathing activities.

### 4.3. Risk Assessment for Drinking Water Consumption

The treatment of river water to produce drinking water aims to remove parasites, bacteria and viruses from the initial matrix, in order to ensure a compliant microbiological quality of drinking water for the consumer. The three drinking water treatment plants, corresponding to sites A, B and C, provide a daily production of 750,000 m^3^ of water for more than four million consumers, representing 99% of the water distributed in the studied area. The risk assessment was focused on the drinking water treatment plant C, which provide the analyzed tap water in this study.

The treatment process used at the drinking water treatment plant C consists in several steps (screening/pumping/pre-ozonation, coagulation/flocculation/decantation, biological sand filtration, ozonation, biological filtration on activated carbon, chlorination and transportation through the pipes). Microorganism removal mainly occurs during the coagulation/flocculation/decantation, biological sand filtration, ozonation and chlorination steps.

Based on the occurrence results, it can be noted that for a daily production of 340,000 m^3^ of drinking water at site C, a maximum of 3.4 × 10^7^ *C. parvum*, 2.0 × 10^8^ *G. duodenalis*, 1.2 × 10^10^ *C. perfringens*, 1.2 × 10^11^ *E. coli* and 2.9 × 10^10^ adenoviruses may enter the treatment plant daily.

For both parasites, maximum concentrations of 0.1 *C. parvum* L^−1^ and 0.6 *G. duodenalis* L^−1^ were taken into account in raw water C. Previous studies reported a minimum physical removal of 4.5 log for parasites (considering the cumulative effect of a conventional coagulation/flocculation process (2.5 log reduction) followed by a slow sand filtration (2 log reduction)) [46]. An additional removal of 0.5 log minimum was attributable to the ozonation step [47,48]. Chlorine disinfection seems to be ineffective at removing parasites from drinking water [49,50], although some studies have demonstrated a possible synergy between ozonation and chlorination for *C. parvum*, whereby the overall removal is increased by about 2 log [46,51].

In the same way, for bacteria, maximum concentrations of 3.4 × 10^1^ *C. perfringens* L^−1^ and 3.6 × 10^2^ *E. coli* L^−1^ were taken into account in raw water C. Previous studies reported a removal of 3 to 3.5 log during the water treatment process [52]. Moreover, for *E. coli*, the directive 98/83/EC advocates an absence in 100 mL.

For adenovirus, a maximum concentration of 8.4 × 10^1^ per liter was taken into account in river water C. It was previously demonstrated that the virus population was reduced during the drinking water treatment with a minimum efficiency of 4 to 5 log [53]. Interestingly, the average cumulative virus reduction did not significantly decrease after ozonation or final chlorination [53]. As a result, a theoretical absence of parasites, bacteria and viruses is expected in the finished water after the treatment process.

To verify the quality of the distributed water supplied from the drinking water treatment plant C, eight tap water samples were tested for each microorganism (1000 L filtered).

Considering a daily consumption of 1 L of tap water per person and that all the tested tap water samples were negatives for each microorganism, the risk was less than 0.02% for both parasites and less than 0.5% for adenovirus.

The absence of microorganisms detected in 1000 L of drinking water taken directly from the tap, taking into account recovery rates and the detection limits of the methods, reinforces the theoretical log-reduction assessment. The effectiveness of the drinking water treatment process to eliminate microorganisms from the river water resource C was thus demonstrated for the tested samples, which guarantees the safety of the consumer. These results also suggest that the circulation of the produced water from the drinking water treatment plant through the 10 km of pipeline to the tap did not cause release of these pathogens from any biofilm in the network.

## 5. Conclusions

In the present study, a two-year monitoring of pathogens and indicators was conducted in three river waters used for recreational activities and as resources for drinking water production, and on a tap water served by a drinking treatment plant fed by one of the three rivers. The influence of land use on microorganism concentrations and health risk assessment in case of river bathing or drinking water consumption were evaluated.

- Overall, considering the seasonality of microorganisms, lower occurrences were found in summer, when the maximum recreational activity occurs. Results showed that lower levels of both parasites (*C. parvum* and *G. duodenalis*) were measured in comparison with *C. perfringens* spores and adenoviruses, the highest concentrations having been found for *E. coli*.
- Land use of the watersheds suggests that microorganism contaminations come from both point and non-point sources. It is known that wastewater treatment plants are the main source of waterborne pathogens, as for human adenoviruses. For *C. parvum* and *G. duodenalis*, leaching of soils from agricultural territories could be also an important input of these parasites. However, a microbial source tracking approach should be the next step in order to confirm these hypotheses.
- The probability of infection for pathogenic parasites in the studied areas in case of recreational activities was lower than 0.5% for adult and child swimmers, in summer. For adenovirus, the risk was the most important, varying from 1 to 42%.
- Taking into account the theoretical efficiency of drinking water treatment plants, and the determined concentrations in the resource, an absence of studied microorganisms is expected in the finished drinking water. The absence of these microorganisms in one thousand-liter samples demonstrated the effectiveness of the drinking water treatment process and an absence of re-contamination from the pipeline.

This study confirms the interest of such sampling campaigns to move towards a more realistic risk assessment approach. Using the ultrafiltration principle to concentrate simultaneously a relevant panel of indicators and pathogens should allow coping with the land use influence on river water and highlight any deficiencies related to drinking water production and/or distribution.
